# Provider Perceptions of Perinatal Mental Healthcare Access

**DOI:** 10.1111/birt.70029

**Published:** 2025-10-31

**Authors:** Susanna Sutherland, Amanda L. Stone, Sarah S. Osmundson

**Affiliations:** ^1^ Department of Physical Medicine and Rehabilitation Vanderbilt University Medical Center Nashville Tennessee USA; ^2^ Department of Obstetrics and Gynecology Vanderbilt University Medical Center Nashville Tennessee USA; ^3^ Department of Anesthesiology Vanderbilt University Medical Center Nashville Tennessee USA

**Keywords:** healthcare access, perinatal mental health, quality improvement, women's mental healthcare

## Abstract

**Background:**

The consequences of untreated perinatal mental health conditions are well‐established, yet fewer than one in five women experiencing perinatal mental health distress receive treatment. Although recommendations for evidence‐based treatment are increasingly widespread, patients and providers still face substantial hurdles to accessing needed services. This study sought to update the literature with a report on providers' perceptions of the demand for and accessibility of mental health services for women in the perinatal period with the goal of pinpointing areas where quality improvement should be implemented.

**Methods:**

An eight‐item, mixed‐methods (i.e., open choice, multiple choice, and open response) questionnaire assessing perceptions of patients' access and barriers to care was sent in a department‐wide email at a large academic medical center. Forty‐six providers completed the survey (*n* = 18 physicians, *n* = 14 nurse midwives, *n* = 13 advanced practitioner nurses, *n* = 2 other providers).

**Results:**

Providers reported pervasive barriers to perinatal mental healthcare and that only occasionally are their perinatal patients able to access appropriate mental healthcare.

**Discussion:**

The findings contribute to the growing body of knowledge regarding access to mental healthcare, ultimately aiming to improve the overall well‐being of women during the perinatal period. The study emphasizes the ongoing critical need for researchers and the healthcare system to recognize and address the persistent challenges faced by obstetric providers, highlighting the pervasive nature of issues in accessing quality perinatal healthcare and underscoring the importance of acknowledging these challenges for justifying increased clinical access, rigorous intervention studies, and policy change.

## Graphical abstract

Study methodology and conclusions: Primary barriers to care and action items for providers.

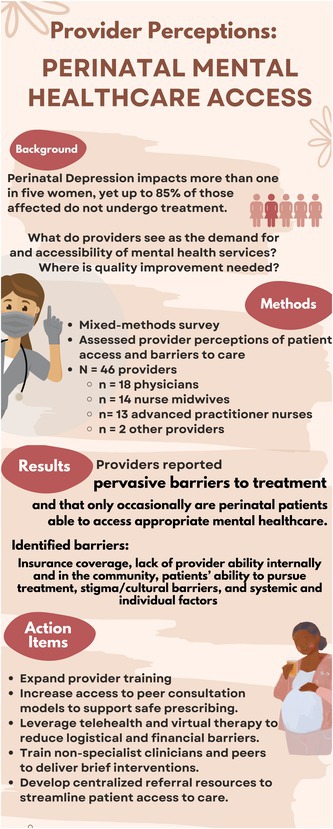

## 1 Introduction

The perinatal period is a critical phase in a woman's life, with the potential for mental health challenges that may have a significant and long‐lasting impact. Perinatal depression (PND), or depressive symptoms that manifest during either pregnancy or the postpartum phase, impacts more than 1 in 5 women, and disproportionately affects women of racial/ethnic minority and low socio‐economic status [1, 2, 3]. PND is associated with increased rates of maternal and infant morbidity and mortality and when left untreated, PND can lead to intergenerational consequences [4, 5, 6]. Despite the well‐documented burden of disease, empirically supported treatment recommendations and a robust literature describing psychological interventions, PND remains woefully undertreated [7, 8]. In 2016, a high‐quality, comprehensive analysis reported that approximately 85% of those experiencing postpartum depression do not undergo treatment at all [7].

Despite the recognized importance of mental healthcare during pregnancy and the postpartum period, access to such services remains elusive. Barriers to perinatal mental healthcare pose significant challenges for women during a biologically and psychologically sensitive period. Affordability is a pervasive issue, as mental health services can be financially burdensome, limiting access for many. Insufficient insurance coverage further compounds the issue, deterring individuals from seeking the necessary care. Additionally, the shortage of qualified providers specializing in perinatal mental health exacerbates the problem, leading to extended wait times and inadequate support.

The field of obstetrics is experiencing significant provider shortages, leading to overworking and burnout, and providers' ability to spend extensive efforts in locating referrals within already‐scarce community‐based mental healthcare is likely diminished [9]. In order to identify targets to improve both the provider experience and patient care, this quality improvement project sought to understand how providers within one of the largest medical centers in the southeastern United States view their local access to mental healthcare referrals. The primary objective of this study was to conduct a current needs assessment for perinatal mental healthcare services in a large academic medical center with the hypothesis that obstetric providers would report both high need for and low access to mental healthcare for individuals during the perinatal period. A secondary objective was to qualitatively discuss opportunities for quality improvement.

## 2 Methods and Materials

### 2.1 Participants

This is a cross‐sectional quality improvement study conducted in November–December 2023 in a large academic medical center in the southeastern United States. The study was submitted to the medical center's institutional review board and was approved (Internal Review Board #231984). Potential respondents (approximately 157 providers; physicians and advanced practice providers) were recruited via Obstetrics and Gynecology and Midwifery and Women's Primary Care departmental listservs, where they were provided with a brief description of and access to a survey of their perceptions of their patients' perinatal mental health needs. Listserv email recipients include clinical faculty and non‐faculty providers, research faculty, and advanced trainees and are largely from the Division of General Obstetrics & Gynecology. The listserv recruitment emails included a message from the study research team providing an overview of and the rationale for the study (as approved by the Internal Review Board) and a link to complete the study. The survey was anonymous and was administered through REDCap [10], a secure, web‐based platform for data capture. Due to the anonymous study nature, only provider designation (e.g., nurse practitioner, certified nurse midwife) and primary department were recorded in lieu of demographic information. Providers were not compensated in any way for participation. There were no exclusion criteria beyond the study's limited distribution to only the Department members with full clinical privileges (and thus not inclusive of junior trainees or clinicians with certifications beneath advanced practice providers).

### 2.2 Questionnaire

Following a review of the literature, we determined that an appropriate extant questionnaire was not available. Therefore, data were collected using a novel 20‐item questionnaire designed to assess obstetric providers' perceptions of the availability of mental healthcare services for women. The survey intended to minimize respondent burden and response bias, given the high demands already placed upon the respondents' profession. The survey contained both qualitative and quantitative items: four multiple option categorical response items, sixteen Likert response items, and one open response area for comments or concerns, totaling 20 items. Survey questions and response options are listed in the Appendix.

### 2.3 Analytic Plan

Descriptive statistics were employed to analyze quantitative data and identify patterns related to obstetric providers' perceptions. In order to evaluate and quantify the magnitude of any significant differences between needs and access, the means of responses were compared with the Wilcoxon signed‐ranks test, a non‐parametric equivalent of the paired *t*‐test. This test provides a *z* score indicating the magnitude of the difference between paired rank data (e.g., Likert scores) and traditional *p* values indicating significance [11].

## 3 Results

Forty‐six participants completed the survey between November 2023 and January 2024. Survey completion took approximately 2–3 min for providers to complete.

### 3.1 Respondents

Providers from two different departments responded to the survey: the Department of Obstetrics and Gynecology and Vanderbilt School of Nursing Faculty Practice Nurse‐Midwives and Primary Care practitioners. Forty‐six respondents of approximately 157 recipients resulted in a response rate of 29%. Respondents identified as obstetric physicians (*n* = 18, 38.3%), certified nurse midwives (*n* = 14, 29.8%) advanced nurse practitioners (*n* = 9, 19.1%), family nurse practitioners involved in the care of pregnant and postpartum women (*n* = 4, 8.5%), and a perinatal geneticist (*n* = 1, 2.1). One provider declined to respond (*n* = 1, 2.1%) (see Table 1).

**TABLE 1:** Clinical role of respondents (*N* = 47).

Clinical role	*n*	%
Obstetric physician	18	38.3
Certified nurse midwife	14	29.8
Advanced practice nurse	9	19.1
Family nurse practitioner	4	8.5
Obstetric geneticist	1	2.1
Declined to respond	1	2.1

### 3.2 Assessment

**FIGURE 1:**
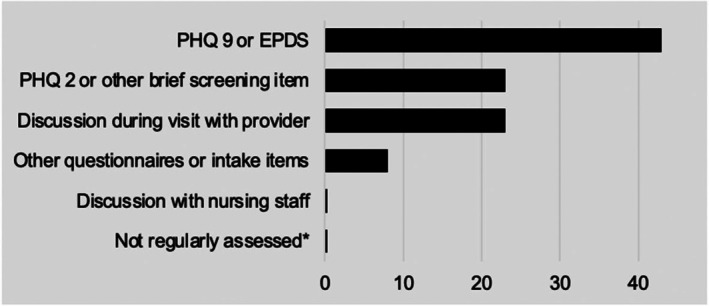
Types of assessment most commonly used. * Mental health is not regularly assessed in my perinatal patients unless they present with a clinical concern or request support.

Perceptions of assessment tools and administration were varied. In response to the question “how is mental health commonly assessed in your patients?,” the majority of respondents (*n* = 43, 91%) endorsed “Patient Health Questionnaire—9 (PHQ‐9) [12] or Edinburgh Postpartum Depression Scale (EPDS),” [13] with almost half (*n* = 23, 49%) also endorsing “Patient Health Questionnaire—2 [14] or Brief Screener” and the same number also endorsing “Discussion with Provider.” Of note, no respondents endorsed that mental health is “not regularly assessed,” or by “other methods” (Figure 1).

**FIGURE 2:**
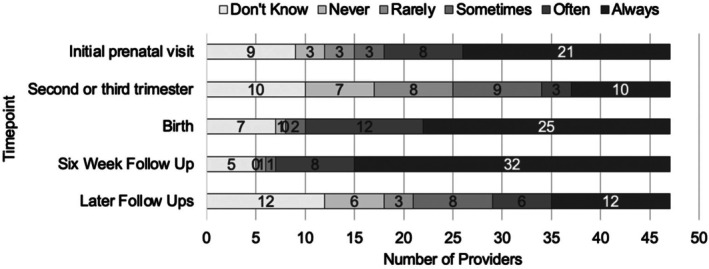
Consistency of assessments responses.

In response to the question “How consistently do your patients complete validated depression screenings around the following time points?” the most common response regarding the initial OB visit, birth, and 6‐week follow‐up appointments was “always,” and regarding the second or third trimester window and later follow‐ups, it was the second most common (exact response numbers and distributions presented in Figure 2).

### 3.3 Patient Needs

Providers reported perceived patient benefits from all services assessed, including psychotherapy (e.g., individual or group therapy) for mental health symptoms, psychopharmacologic treatment for mental health symptoms, more education about mental health symptoms and/or coping skills, and complementary or alternative treatments for mental health concerns (e.g., yoga or movement classes, acupuncture), with “often” as the modal response for all items. Item response frequencies are reported in Table 2. Substantial and significant discrepancies between perceptions of patients' needs and access were identified across all assessed treatment modalities (Table 2). For example, in response to the prompt “Indicate how often you think your patients would benefit from the following service: psychotherapy (individual or group) for mental health symptoms” the median and modal (*n* = 20 participants) response was “often,” as was the average response (*x̄* = 4.11). However, in response to the paired prompt “Indicate how often you think your patients have timely and affordable access to psychotherapy (individual or group) for mental health symptoms” the median and modal (*n* = 23 participants) response was “rarely,” as was the average response (*x̄* = 2.13), a statistically significantly poorer response (*z* = −5.99, *p* < 0.001). The smallest discrepancy, though still highly significant (*p* = 0.001), was between needs of and access to psychopharmacologic treatment.

**TABLE 2:** Needs and access to treatment options.

Treatment options	Indicate how often you think your patients *would benefit* from the following services	Indicate how often you think your patients *have timely and affordable access to*…	*z*	*p*
	Mean (SD); median item	Mean (SD); median item		
Psychotherapy (individual or group) for mental health symptoms	4.11 (0.49); often	2.13 (0.40); rarely	−5.99	< 0.001
Psychopharmacologic treatment for mental health symptoms	3.84 (0.60); often	3.22 (0.85); sometimes	−3.23	0.001
More education about mental health and/or coping skills	4.33 (0.63); often	2.50 (0.66); rarely	−5.73	< 0.001
Non‐pharmacologic treatment for physical health concerns (e.g., yoga, therapy, acupuncture, movement classes, etc.)	4.13 (0.69); often	2.17 (0.64); rarely	−5.72	< 0.001
Complementary treatments for mental health concerns (e.g., yoga or movement classes, acupuncture)	4.00 (0.84); sometimes	2.09 (0.66); rarely	−5.52	< 0.001

*Note:* Response options: 1 (never), 2 (rarely), 3 (sometimes), 4 (often), 5 (always).

### 3.4 Access

**FIGURE 3:**
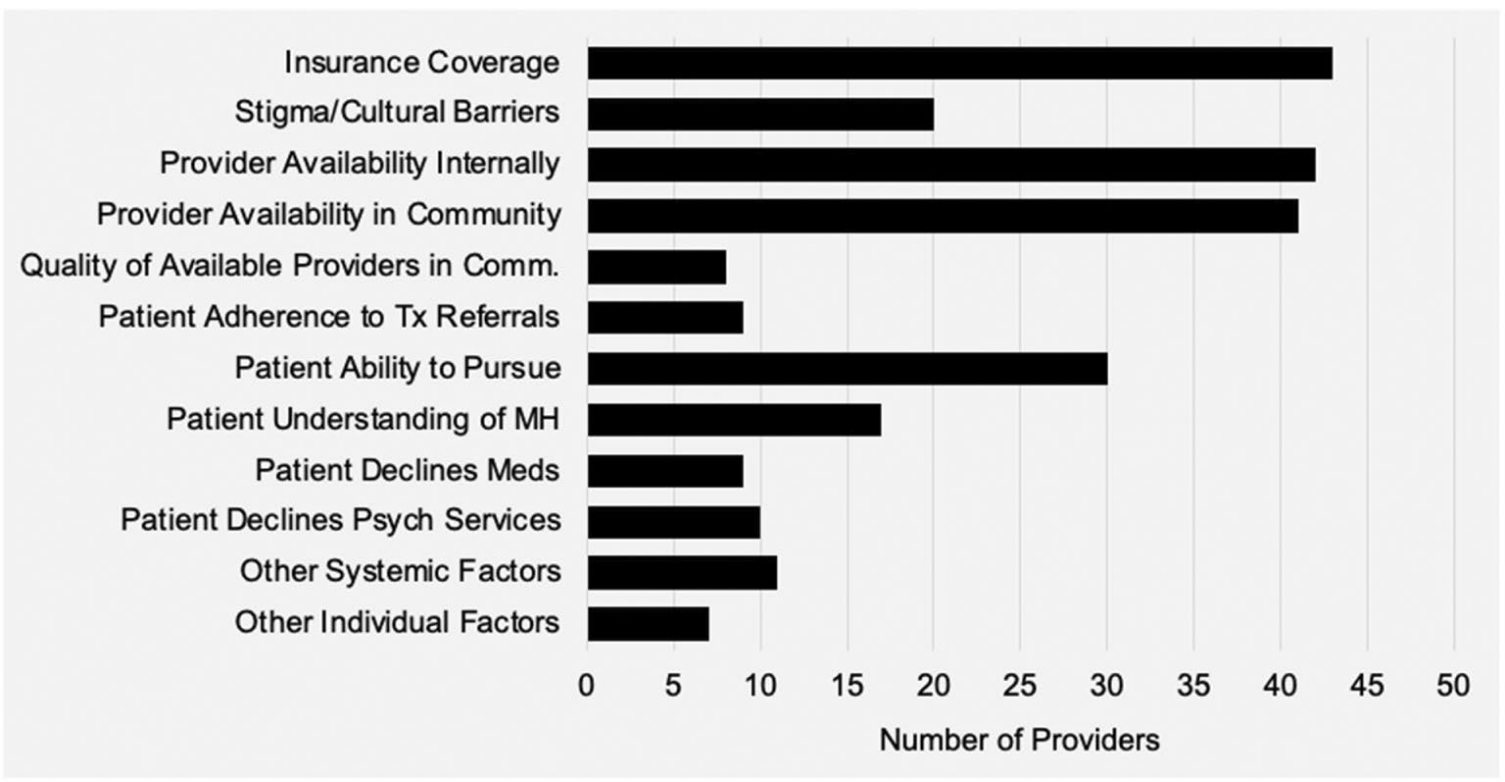
Provider perceptions of barriers to patient treatment.

The most commonly perceived barriers to patient mental healthcare included lack of insurance coverage (91% of respondents), provider availability within the medical center (89% of respondents) and the community (87% of respondents), and patients' ability to pursue care (64% of respondents). None of the listed potential barriers were endorsed by fewer than 10% of respondents (Figure 3).

When asked whether providers felt that their “perinatal patients are generally able to access the mental healthcare they need,” all respondents indicated either only “sometimes” (*n* = 21, 45%) or “rarely” (*n* = 26, 55%).

### 3.5 Qualitative Responses

In total, 22 (47%) survey participants elected to include a comment or concern, and all comments generally addressed access‐related concerns. The most commonly listed items regarded lack of providers accepting referrals, particularly for patients unable to pay out of pocket, and even more so for those receiving state‐funded medical coverage. Seven respondents referenced the significant time lag between referrals and provider openings or long waitlists for care in the community; five respondents specifically noted that there are very few providers that accept Medicaid and relatively few that accept insurance at all. Providers reported that for the few mental health clinicians that do accept insurance, services are still not timely. For example, one participant responded, “The community mental health resources that take Medicaid tend to have long waiting lists, high staff turnover, and can be difficult for our patients to navigate.” Additional representative responses are reported in Table 3.

**TABLE 3:** Representative examples of qualitative responses.

“Peri‐ and postpartum MH [mental health] disorders affect the entire family and can escalate very quickly. It is so frustrating to be told there is no availability for XX weeks!”—Family Nurse Practitioner
“We do so much depression screening but have so few places to refer patients to for counseling/treatment, and long waits for services.”—Certified Nurse Midwife
“Women are suffering from mental health [problems]. Access is a major barrier especially for Medicaid and Coverkids [a state‐funded program that provides obstetric coverage to patients who lack U.S. resident status] recipients.”—Advanced Practice Nurse
“There are such limited resources available for many of our patients with mental health needs often due to insurance/financial reasons. I think anything that could be offered would be a huge step.”—MD
“[What would be most helpful is] timely referral since pregnancy is a highly stressful, but limited duration time, my patients often can't afford to wait months for an appointment.”—MD

## 4 Discussion

This study confirmed an ongoing high need for and significant barriers to accessing perinatal mental healthcare at a tertiary academic medical center. Although most providers reported using standardized tools to assess for perinatal mental health symptoms, their patients were rarely able to access the mental healthcare they needed. This highlights a substantial gap in evidence‐based care as the benefits of systematic screening rely on the ability to connect individuals who screen positive for depression or anxiety with appropriate interventions.

The majority of survey respondents indicated that their patients would benefit from access to a variety of services to support their mental health including psychotherapy, increased opportunities for education about perinatal mental health, psychiatric services, and nonpharmacologic interventions for perinatal health. The high need for access to services to support mental health in this population is understandable given both the prevalence of perinatal mood and anxiety disorders and the fact that the perinatal period is a time of inherent change.

In contrast, the finding that almost all respondents employed within a major medical center reported that their patients can only “rarely” or “sometimes” access the mental healthcare they need, despite the significant attention brought to these issues over the past decade, is less understandable [7]. Ongoing systemic barriers such as insurance access and reimbursement, shortages of mental health providers trained in perinatal care, and lack of accessible integrated care models have likely hindered progress in addressing the gaps between perinatal mental healthcare needs and connection to services [15, 16, 17]. The political climate may also exacerbate barriers to perinatal healthcare. The current study took place in a state with lawmakers pursuing work requirements for low‐income parents and forfeiting federal opportunities to expand Medicaid coverage, resulting in heightened gaps in coverage [18]. These barriers collectively contribute to suboptimal referral patterns, often resulting in delayed or inadequate interventions.

Facilitators to care access have been studied extensively and reviewed elsewhere (e.g., Webb et al. [19]; Browne et al. [20]) and include increased mental health education for providers and patients, ongoing conversation between providers and patients about mental health, and a continuity of care for patients. Routinely conducting standardized assessment of perinatal mental health symptoms can help facilitate these conversations between providers and patients. Results from the current study indicated most providers are commonly relying on well‐validated measures (e.g., PHQ‐9, EPDS), and that these measures are almost always administered at the timepoints recommended by the American College of Obstetrics and Gynecology (ACOG; i.e., initial prenatal visit, later in pregnancy, and postpartum visits) [21]. There was more uncertainty in the current study around measures administration during additional timepoints such as the second or third trimester of pregnancy or additional postpartum visits. This may represent less standardization of care during these visits or an artifact of our questionnaire design as providers may have perceived overlap between the initial patient visit and the second or third trimester.

Based on provider feedback from the current study, additional training in basic perinatal psychopharmacology for obstetric providers would be beneficial for facilitating care. Two potential responses to this need have been implemented in other healthcare systems: increased administrative support for additional training in psychopharmacological treatment, and formalized access to peer consultation. The former may be executed with protection of providers' already strained time for intensive workshops or additional funding to extend providers' scope of practice (e.g., coursework). The latter is already being implemented and expanded in some locations. For example, the Massachusetts Child Psychiatry Access Program for Moms (MCPAP) was created in 2004 and has been continuously expanded, and provides support for primary care providers in managing their patients' mental health as well as providing extensive support for patients in accessing mental healthcare [22]. The MCPAP for Moms phone consultation line is free and accessible to healthcare workers who have questions about managing their patients' medication during the perinatal period, empowering primary care and obstetric providers to safely expand their scope of practice under specialized supervision. This service is funded by both state government and commercial insurance companies and evidence demonstrates its low cost relative to the relieved burden on the healthcare system.

This study, while contributing valuable insights, is constrained by the nature of a quality improvement project. Firstly, reliance on preliminary qualitative data from a convenience sample limits generalizability. While quantitative items did provide an opportunity to explore summary statistics, a more statistically rigorous approach might have allowed for inferential analysis. Further, while provider perceptions of access have historically been reported, this literature is predominantly qualitative in nature, and novel measures described in academic journals are rarely published in full [23]. Thus, this study was limited by the use of a questionnaire that has not yet been formally validated or studied for reliability; this is an inherent weakness and presents an opportunity for future methodological work. In addition, this study reports *provider* perceptions, which may differ from objective access metrics. There is a need to triangulate provider perspectives with objective, system‐level data (e.g., by use of “secret shopper” [24] or database methodology) in the future in order to further tailor recommendations.

Given the location of the study in a relatively underserved state, the findings may not extend to states with more comprehensive perinatal healthcare coverage. Conversely, respondents were all employees of a large academic medical system located in a mid‐size city, and may have greater access than providers in even more underserved areas. Future research endeavors should aim to overcome these limitations by incorporating larger and more diverse samples, as well as utilizing a mixed‐methods approach for a more comprehensive understanding of the phenomenon under investigation.

### 4.1 Future Directions

The most recent report of the U.S. Preventive Services Task Force regarding PND specifically solicited research examining interventions that are both more accessible for patients and less burdensome on the healthcare system [25].

Addressing systemic challenges requires a comprehensive approach, involving policy changes, increased mental health awareness, and efforts to expand the availability of affordable, specialized care for perinatal mental health. Historically, the U.S. healthcare system has addressed issues of physical and behavioral health separately, resulting in distinct training paths for providers of each domain. However, a growing push to merge services has shown “consistently successful” improved patient outcomes [26]. Across types of healthcare settings, this integration may manifest in different ways. For example, health centers may begin to include behavioral healthcare providers within their physical healthcare clinics [27]. Alternatively, healthcare systems can invest in psychiatry consult‐liaison services or patient navigators that provide triage and referral services for obstetric patients. In other settings, primary care providers may seek out additional training in providing mental healthcare screening, non‐specialist interventions (e.g., psychoeducation), or initiating psychopharmacological treatment. However, each of these solutions requires significantly more financial investment and policy change by healthcare administration, both on local setting and more universal, governmental levels.

Other, possibly more feasible, solutions may respond to pragmatic barriers to perinatal mental healthcare. Specialized behavioral healthcare is particularly inaccessible for the 169 million people in the United States living in Mental Health Professional Shortage Areas, and behavioral healthcare provider shortages are expected to worsen [28]. Expanding telemedicine access offers the potential to extend the reach of behavioral healthcare with the added benefit of addressing barriers such as the need for childcare during appointments and the time and financial burden of traveling to appointments [29]. Another solution entails training non‐specialist clinicians (e.g., obstetric or public health nurses) or non‐clinician peers (women with a history of PND but currently remitted) to deliver evidence‐based care to women in need [30]. This solution addresses both pragmatic barriers and cultural barriers to care.

In service of summarizing and synthesizing the preceding discussion, we propose that the following specific, actionable strategies may serve the obstetric community. First, standardize and expand screening measures, routinely administering validated screening tools (e.g., PHQ‐9 or EPDS) at timepoints beyond ACOG's current guidelines (e.g., at the 20‐ and 32‐week appointments, at additional postpartum follow‐up visits sometimes required for patients with complicated or surgical deliveries, at the first subsequent annual well‐woman appointment). Measures can be integrated into existing patient workflows through electronic medical records to track trends or automatically triage for concerning responses. Departments can offer protected time or CME credit for providers to complete mental health training modules or attend in‐service trainings on PMADs, trauma‐informed care, or basic perinatal psychopharmacology. Targeted education or provider toolkits may help address knowledge gaps and optimize referrals to reduce this barrier. Existing psychiatric consultation lines can serve as models to replicate (e.g., MCPAP), providing service to rural areas or areas otherwise not serviced by specialists. Telehealth visits and/or options for virtual therapy will offer increased accessibility for patients and at lower costs to providers, and these should be covered by insurance and available outside of work hours. Centralized provider referral lists can be generated and made available to all providers and integrated into existing workflows for delivery to patients. Finally, obstetric nurses or clinical assistants can be trained in brief evidence‐based interventions (e.g., psychoeducation, behavioral activation) or limited components of cognitive‐behavioral therapy.

The potential impact of improved access to perinatal mental healthcare is profound. Perinatal depression is vastly undertreated and the consequences are considerable for women, children, and society as a whole. Poggi Davis and Narayan [31] describe aspects of the perinatal time as a sensitive period. First, it is a period of rapid change: mothers experience vast physiological changes across almost all organ systems, including the HPA axis, and the fetus develops at a meteoric rate. As women transition into a self‐concept as a mother and prepare for parenthood, thoughts about or effects of historical adverse events may reappear. Further, pregnancy is a period of intense social change, and stressful life events (e.g., extreme poverty, distress with romantic partners) may increase and convey additional risk. In addition, it is well established that maternal stress during gestation is teratogenic. Fetal programming of maternal stress or mental health disorder can have significant and lifelong impacts on offspring's physical, cognitive, and emotional development [5]. Finally, and possibly most importantly, this period is also a window where positive changes may result in adaptation and resiliency for both mother and offspring. Further, some mothers are particularly motivated to change for the future of their child, and thus, even with pre‐existing mental health risks, this window remains an opportunity to capitalize on a crucial point of intervention [31].

## 5 Conclusion

Results of the present study highlighted the ongoing gap between perinatal mental healthcare needs and access to services which impacts both patients and obstetric care providers. It is imperative for academia and healthcare systems to recognize the ongoing inherent challenges faced by obstetric providers in supporting their patients' care. Acknowledging these challenges is essential in the justification of increasing clinical access, rigorous study of intervention implementation, and laying the groundwork for policy change.

## Conflicts of Interest

The authors declare no conflicts of interest.

## Data Availability

The data that support the findings of this study are available from the corresponding author upon reasonable request.
